# Reliability of comorbidity indices as predictive indicators for frequent severe chronic obstructive pulmonary disease exacerbations

**DOI:** 10.5578/tt.202401833

**Published:** 2024-03-26

**Authors:** Deniz DOĞAN MÜLAZİMOĞLU, Bilge BİLGİN, Sümeyye AYÖZ, Fatma ARSLAN, Elif ŞEN

**Affiliations:** 1 Department of Chest Diseases, Ankara University Faculty of Medicine, Ankara, Türkiye; 2 Department of Chest Diseases, Sakarya Research and Training Hospital, Sakarya, Türkiye

## Abstract

**ABSTRACT**

**
Reliability of comorbidity indices as predictive indicators for frequent severe chronic obstructive pulmonary disease exacerbations
**

**Introduction:** *The relationship between comorbidities and chronic obstructive pulmonary disease (COPD) is two-sided. As the number of comorbidities increases, frequency of acute exacerbations of COPD (AECOPD) conse- quently increases. Comorbidity indices can be used to evaluate comorbidities while managing COPD patients. We aimed to compare comorbidity indices such as the Charlson comorbidity index (CCI), comorbidities in COPD index (COMCOLD) and COPD specific comorbidity test (COTE) regarding exacer- bation frequency.
*

**Materials and Methods:** *Participants hospitalized for AECOPD were included in this bidirectional case-control study. Exacerbation severity, frequency, fur- ther exacerbations over a one-year follow-up period and CCI, COMCOLD, and COTE scores were recorded. High and low comorbidity groups were com- pared regarding AECOPD frequency, severity, and further exacerbations.
*

**Results:** *Ninety-two patients were enrolled. The frequency of AECOPD was significantly higher in high-comorbidity groups (p= 0.026 for CCI; 0.015 for COTE; 0.012 for COMCOLD) than that in low-comorbidity groups. Severe AECOPD was significantly higher in all high-comorbidity groups according to the indices. Median number of exacerbations during the one-year follow-up period was significantly higher in the high-comorbidity groups defined by CCI [0 (0-4) vs. 1 (0-4), p< 0.001 and COMCOLD 0 (0-4) vs. 1 (0-3), p= 0.007].
*

**Conclusion:** *Comorbidities are among the most important risk factors for AECOPD. Managing comorbidities begins with their identification, followed by appropriate interventions. Therefore, using at least one comorbidity index during assessment ensures that comorbidities are not overlooked during diag- nostic and therapeutic processes. CCI, COTE, and COMCOLD comorbidity indices can be used in predicting COPD exacerbations.
*

**Key words:** *Acute exacerbation; comorbidity index; chronic obstructive pulmonary disease
*

**ÖZ**

**
Ağır kronik obstrüktif akciğer hastalığı alevlenmelerinin sıklığını öngörmede komorbidite indekslerinin güvenilirliği
**

**Giriş:** *Komorbiditeler ile kronik obstrüktif akciğer hastalığı (KOAH) arasındaki ilişki çift yönlüdür. Eşlik eden hastalık sayısı arttıkça, KOAH'ın akut alevlenmelerinin (AECOPD) sıklığı artar. KOAH hastalarının yönetiminde eşlik eden hastalıkları değerlendirmek için komorbidite indeksleri kullanılabilir. Charlson komorbidite indeksi (CCI), KOAH’daki komorbidite indeksi (COMCOLD) ve KOAH özgül komorbidite testi (COTE) gibi komorbidite indekslerini AECOPD sıklığı açısından karşılaştırmayı amaçladık.
*

**Materyal ve Metod:** *AECOPD nedeniyle hastaneye yatırılan katılımcılar bu iki yönlü vaka kontrol çalışmasına dahil edildi. Alevlenme şiddeti, sıklığı, bir yıllık takip süresince alevlenme ve CCI, COMCOLD ve COTE puanları kaydedildi. Yüksek ve düşük komorbidite grupları, AECOPD sıklığı, şiddeti ve takipte alevlenme açısından karşılaştırıldı.
*

**Bulgular:** *Doksan iki hasta dahil edildi. AECOPD sıklığı yüksek komorbidite gruplarında (CCI için p= 0,026; COTE için 0,015; COMCOLD için 0,012) düşük komorbidite gruplarından önemli ölçüde yüksekti. İndekslere göre tüm yüksek komorbidite grupların- da şiddetli AECOPD önemli ölçüde daha fazlaydı. Bir yıllık takip süresince alevlenme sayısının ortanca değeri CCI ile belirlenen yüksek komorbidite gruplarında [0 (0-4)’e karşı 1 (0-4), p< 0,001 ve COMCOLD ile 0 (0-4)’a karşı 1 (0-3), p= 0,007] önemli ölçüde daha yüksekti.
*

**Sonuç:** *Komorbiditeler, AECOPD için en önemli risk faktörlerinden biridir. Eşlik eden hastalıkların yönetimi, tanıdan başlayarak uygun müdahaleleri takip ederek başlar. Bu nedenle, en az bir komorbidite indeksi kullanmak, tanısal ve terapötik süreçlerde eşlik eden hastalıkların göz ardı edilmemesini sağlar. CCI, COTE ve COMCOLD, KOAH›da alevlenmelerle ilişkili komorbiditeleri değerlendirmek için faydalı araçlardır.
*

**Anahtar kelimeler:** *Akut alevlenme; komorbidite indeksi; kronik obstrüktif akciğer hastalığı
*

## INTRODUCTION

Acute exacerbation of chronic obstructive pulmonary disease (AECOPD) is defined as the acute worsening of respiratory symptoms necessitating additional therapy (1,2). Exacerbations are not only the leading cause of mortality in patients with COPD, but also tremendously impact the patient’s quality of life (3,4). During exacerbations, an increase in systemic inflammation accompanied by elevated oxidative stress is observed. Consequently, this results in an increase in the number of comorbidities. Exacerbations can also worsen conditions such as coronary heart disease, osteoporosis, and malnutrition (5-7). However, exacerbations do not only worsen comorbidities, but are also impacted by comorbidities: the relationship between comorbidities and exacerbations is direct and two-sided. In the Evaluation of COPD Longitudinally to Identify Predictive Surrogate Endpoints (ECLIPSE) study, severe bronchial obstruction was insufficient to explain COPD exacerbation (8). In order to understand the reasons for frequent exacerbations, a cluster analysis was performed, and two groups with frequent exacerbations were classified: severe obstruction syndrome and moderate obstruction syndrome with comorbidities (9,18). Anxiety and depression, metabolic syndrome, gastroesophageal reflux disease, coronary artery disease, diabetes, and venous thromboembolism are some comorbidities known to

increase AECOPD frequency (10-16). The number of comorbidities is directly related to exacerbation frequency, risk of hospitalization, quality of life, annual costs of disease, and mortality in COPD (17,18). COPD often coexists with other diseases that can significantly affect its prognosis. Comorbidities are frequently observed in patients hospitalized for COPD exacerbations (19,20). Moreover, frequent exacerbations are directly related to COPD severity and previous exacerbation history.

Therefore, comorbidity indices should be systematically used to prevent comorbidities co-existent with COPD from being overlooked. There are indices for multisystemic evaluation, such as the Charlson comorbidity index (CCI), and those specific to COPD patients, such as the COPD specific comorbidity test (COTE) and comorbidities in COPD index (COMCOLD). Those comorbidity indices are widely used. However, which index is more reliable as a predictive indicator for frequent severe COPD exacerbations is unknown.

In the present study, we aimed to investigate whether high comorbidity levels compared to low comorbidity levels, defined using different comorbidity indices (CCI, COTE, and COMCOLD), were associated with frequent severe exacerbations in the year preceding index hospitalization and future exacerbations in the year following index hospitalization in patients with severe COPD exacerbations.

## MATERIALS and METHODS

**Study Population and Data Collection**

We included patients admitted to the pulmonary diseases department of our institution with AECOPD and hospitalized for a six-month period. Patients who did not provide voluntary informed consent were excluded. The study was approved by the Human Research Ethics Committee of Ankara University Faculty of Medicine (05-305-18).

Demographics, smoking history, COPD assessment test (CAT) score, mMRC dyspnea score, and spirometry tests that were obtained during the stable period in the previous year, as well as arterial blood gas (ABG) test results, use of respiratory support devices, comorbidities, CCI, COTE scores, COMCOLD, Global Initiative for Chronic Obstructive Pulmonary Disease (GOLD) classification of patients, and frequency of exacerbations in the previous year were recorded on the day of hospitalization for AECOPD. In addition, patients were followed-up for one year from the day they were enrolled, and the exacerbation data collected.

## Study Design

Patients admitted to the hospital for AECOPD were asked about exacerbations experienced in the year preceding the date of admission for this cross- sectional study via face-to-face interviews. A non- severe COPD exacerbation is defined as the absence of the need for hospitalization and respiratory failure.

The primary endpoint was the number of AECOPD episodes experienced throughout the year, regardless of severity, before index admission. One of the secondary endpoints was the presence of at least one severe AECOPD episode in the previous year. Severe AECOPD was defined as an exacerbation that required hospitalization for management. The other secondary endpoint was the number of AECOPD episodes during a one-year period from the preceding admission, regardless of severity.

## Comorbidity Indices

The CCI was proposed in 1987 and includes 15 chronic diseases, including coronary heart disease, chronic heart failure, chronic lung disease, peripheral vascular disease, cerebrovascular disease, dementia, diabetes, systemic hypertension, liver disease, renal

disease, cancer, metastatic solid neoplasm, connective tissue disease, HIV, peptic ulcer disease, leukemia, and lymphoma (Table 1) (21). This index is applied in a wide spectrum of chronic and systemic diseases, apart from COPD. The CCI is used to predict the risk of mortality within one year of hospitalization for patients with comorbid conditions. A high comorbidity level in the CCI index is defined as a score ≥2 (22).

The COTE index was defined in 2012, and is based on 12 comorbidities: anxiety, coronary heart disease, heart failure, cancer, liver cirrhosis, atrial fibrillation/ flutter, diabetes with neuropathy, pulmonary fibrosis, and peptic ulcer disease (Table 1) (23). This is a COPD-specific index that predicts the survival of COPD patients. A high comorbidity level in the COTE index is defined as a score of ≥4 points (23).

The COMCOLD index was proposed in 2014 (24). The COMCOLD index is based on five comorbidities: depression, anxiety, peripheral artery disease, cerebrovascular disease, and symptomatic heart disease (Table 1). This index is used in COPD patients to determine the effect of comorbidities that most profoundly affect the health status and mortality. A high comorbidity level is defined as a score of ≥4 points.

## Diagnostic Procedures

Spirometry: Expiratory flow rates were measured at rest using the Vmax Encore 229 Pulmonary Function (Sensor Medics, Yorba Linda, CA, USA) according to American Thoracic Society/European Thoracic Society recommendations (25). Flow-volume and volume-time curves were interpreted, and forced vital capacity (FVC), forced expiratory flow in one

second (FEV1), FEV1/FVC, forced mid-expiratory flow (FEF 25-75%), and peak expiratory flow (PEF) were

measured.

## Arterial blood gases

Arterial blood gas analysis was performed if the patient’s peripheral capillary oxygen saturation (SpO2) reading was <94% on pulse oximetry or if they exhibited symptoms of hypercapnia. PaO2, PaCO2, pH, and oxygen saturation (SaO2) in arterial blood were recorded.

**Table:** 

**Table 1.** Charlson, COTE and COMCOLD indices			
**CCI**		**COTE**	**COMCOLD**
Depression			6
Anxiety		6*	4
Peripheral artery disease	1		3
Cerebrovascular disease			3
Symptomatic heart disease (Coronary heart disease and/or heart failure)	1	1	3
	1	1	
Lung, breast, esophageal or pancreatic cancer	2	6	
All other cancers		2	
Metastatic solid tumor	6		
Liver cirrhosis (COTE) Moderate or severe liver disease (CCI) Mild liver disease (CCI)	3 1	2	
Atrial fibrillation/flutter		2	
Diabetes with neuropathy (COTE) with chronic complications (CCI) without chronic complications (CCI)	2	2	


	1		
Pulmonary fibrosis		2	
Congestive heart failure			
Gastric/duodenal ulcer	1	1	
Myocardial infarction	1		
Dementia	1		
Chronic pulmonary disease	1		
Connective tissue disease	1		
Hemiplegia or paraplegia	2		
Renal disease	2		
AIDS/HIV	6		
Leukemia	2		
*Only for female			
The indices and weights of comorbidities are given in the table. Maximum score for CCI: 34, for COTE: 25, for COMCOLD: 19. CCI: Charlson comorbidity index, COTE: Chronic obstructive lung disease specific comorbidity test, COMCOLD: Comorbidities in chronic obstructive lung dis- ease.			

## Statistical Analysis

Data were analyzed using IBM SPSS Statistics version

22.0 (IBM Corp., Armonk, NY,). Variables were investigated using visual (histograms and probability plots) and analytical methods (Kolmogorov-Smirnov/ Shapiro-Wilk test) to determine normal distribution. Frequency analyses were performed for categorical variables of the entire sample, and mean or median values were calculated for continuous variables. Sociodemographic, radiologic, clinical, and

laboratory data of all patients were compared using the Chi-square test for categorical variables and Student’s t-test or Mann-Whitney U test according to the distribution pattern for continuous variables. A 5% type-I error level was used to indicate statistical significance.

## RESULTS

A total of 92 patients [median age= 70 (range= 49–88) years] met the inclusion criteria and were

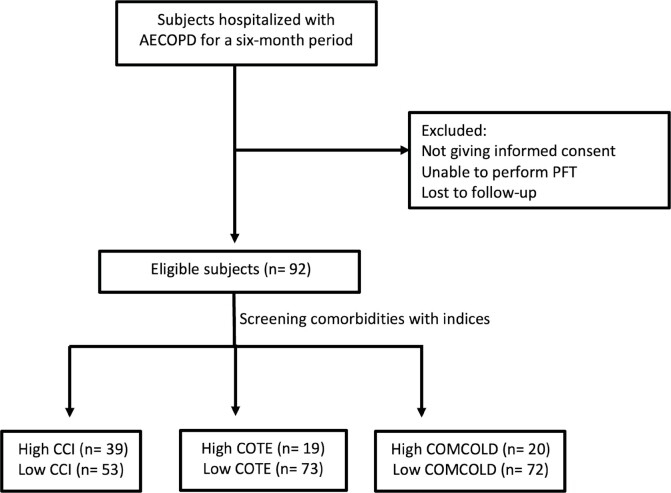

**Figure 1.** Flowchart of the study.

AECOPD: Acute exacerbations of chronic obstructive lung disease, PFT: Pulmonary function test, CCI: Charlson comor- bidity index, COTE: Chronic obstructive lung disease specific comorbidity test, COMCOLD: Comorbidities in chronic obstructive lung disease.

enrolled in the study (Figure 1). Among the patients, 13% were females and 87% were males. Regarding smoking history, 2.2% never smoked, 75% were ex-smokers and 22.8% were current smokers. Median CAT score was 18 (range= 6-40). Regarding GOLD classification of the patients just before this exacerbat ion, 13% of COPD patients were classified as GOLD A, 21.7% as GOLD B, 8.7% as GOLD C, and 56.5%

as GOLD D. Treatment during the stable period was recorded. Seventy-three patients used long-acting bronchodilators, while the rest (n= 19) used short- acting bronchodilators. Among all patients, 48.9% received long-term oxygen therapy (LTOT), while 16.3% underwent non-invasive mechanical ventilation (NIMV).

There was no correlation between age and frequency of AECOPD (p= 0.937); however, there was a weak negative correlation between age and ICU admission (p= 0.028, Spearman’s rho= -0.229). The participants’ sex did not affect the frequency of AECOPD (p=

0.327) or its severity (outpatients p= 0.068, emergency department admissions p= 0.340, hospitalizations p= 0.897, ICU admissions p= 0.952). History of smoking (measured in pack-years) did not correlate with the frequency of AECOPD but correlated positively with ICU admission (p= 0.009, Spearman’s rho= 0.273). Neither frequency nor severity of AECOPD differentiated smoking cessation (p= 0.849, p= 0.701, p= 0.588, p= 0.373, p= 0.514, respectively). Although the need for LTOT was statistically significant for the frequency of AECOPD (p< 0.001), the need for NIMV was not (p= 0.108).

A negative correlation was observed between the

frequency of AECOPD, FEV1%, and FEV1/FVC (p= 0.001, Spearman’s rho= -0.376 and p= 0.045, Spearman’s rho= -0.224; respectively). In contrast, there was no correlation between FVC and AECOPD frequency (p= 0.106). FEV1% and ICU admission due to AECOPD exhibited a strong correlation (p< 0.001, Spearman’s rho= -0.448).

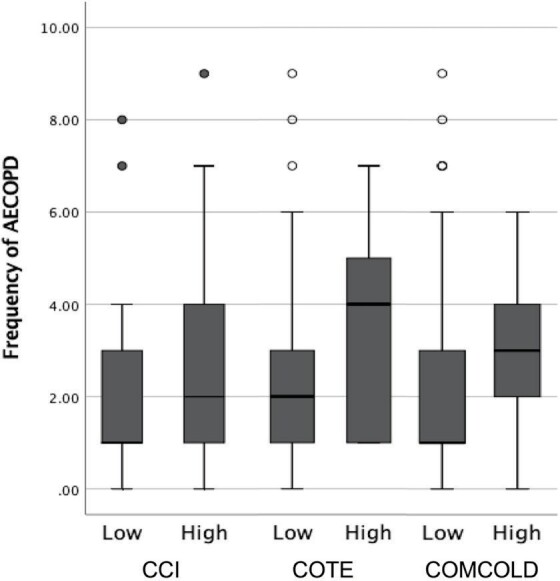

**Figure 2.** Relationship between exacerbation frequency and comorbidity groups according to incides

AECOPD: Acute exacerbations of chronic obstructive lung dis- ease, CCI: Charlson comorbidity index, COTE: Chronic obstruc- tive lung disease specific comorbidity test, COMCOLD: Comorbidities in chronic obstructive lung disease.

Arterial blood gas analysis was performed on the day of admission for AECOPD. Although pH, PaCO2, and PaO2 were not correlated, oxygen saturation was negatively correlated with the frequency of AECOPD (p= 0.007, Spearman’s rho= -0.289). However, PaCO2 and the need for ICU admission had a strong correlation (p= 0.001, Spearman’s rho= -0.367).

The frequency of AECOPD was significantly higher in the high-comorbidity group (p= 0.026 for CCI, 0.015 for COTE, and 0.012 for COMCOLD) (Figure 2). Correlation analysis was conducted to obtain an estimation of the effect of the indices on AECOPD. There was a weak correlation between the frequency

of AECOPD and CCI, COTE, and COMCOLD scores (p= 0.017, Spearman’s rho= 0.249; p= 0.0, Spearman’s

rho= 0.243; p= 0.005, Spearman’s rho= 0.292, respectively). The incidence of severe AECOPD was significantly higher in all of the high-comorbidity groups than that in the low-comorbidity groups according to the indices (Table 2). Median number of exacerbations during the one-year follow-up period was significantly higher in the high-comorbidity group than that in the low-comorbidity group, as defined by CCI [0 (0.4) vs. 1 (0-4), p< 0.001 and

COMCOLD 0 (0-4) vs. 1 (0-3), p= 0.007] scores.

However, there was no difference between comorbidity groups as defined by COTE [0 (0-4) vs. 1 (0-2), p= 0.058].

## DISCUSSION

We investigated the relationship between comorbidities and the frequency of AECOPD. Comorbidities assessed using CCI, COTE, and COMCOLD indices were found to be important in estimating both the frequency and severity of AECOPD. However, the power of the indices did not differ in this regard.

Comorbidities are well-known risk factors for COPD exacerbation, need for hospitalization, prolonged hospitalization, and mortality (19,20,22,23). Therefore, managing and treating only COPD is not sufficient to reduce the risk of AECOPD and increase the patient’s quality of life. Managing comorbidities is inherent in managing COPD. However, this is only possible when comorbidities are identified by physicians. The role of these indices in the systematic recognition of comorbidities was presented herein.

The CCI is a general index that has been studied not only for COPD but also for many other chronic diseases. It is associated with mortality and hospital

**Table:** 

**Table 2.** Comparison of exacerbation severity and scores of the indices			
	**Severe exacerbation**	**Non-severe exacerbation**	**p**
CCI- Low	16 (41.0)	23 (58.9)	
			**0.028**
CCI- High	34 (64.1)	19 (35.8)	
COTE- Low	34 (46.5)	39 (53.4)	
			**0.004**
COTE- High	16 (84.2)	3 (15.7)	
COMCOLD- Low	35 (48.6)	37 (51.3)	
			**0.036**
COMCOLD- High	15 (75)	5 (25)	
CCI: Charlson comorbidity index, COTE: Chronic obstructive lung disease specific comorbidity test, COMCOLD: Comorbidities in chronic obstructive lung disease. Results were given as n (%).

readmission in COPD patients (26,27). In addition, we found that CCI was associated with both the frequency and severity of AECOPD. It is a large-scale index, and recognizing various comorbidities and effectively managing them can impact the course of COPD. Many comorbidities evaluated in patients with COPD are present in CCI. This could be the reason for its high reliability in estimating anticipated exacerbations.

COTE is a specific index for the survival of patients with COPD. Higher COTE index scores have been associated with increased mortality (23). The relationship between COTE and the number of exacerbations was assessed in the present study, and higher levels of COTE were observed in patients with frequent and severe AECOPD compared to those in patients without frequent and severe AECOPD. In a study comparing CCI and COTE, the reliability of the indices at predicting mortality was similar (28). Moreover, we also found that the reliability of indices in predicting exacerbation frequency and severity were similar.

COMCOLD is a much more specific index than CCI that aims to screen for comorbidities that affect patients with COPD the most. This index reflects the patient’s health status (24). One study identified no correlation between COMCOLD scores and frequent exacerbations (GOLD C-D) (29). In contrast, the scores of the high-comorbidity group (GOLD B-D) were higher than those of the other groups (29). However, the frequency and severity of acute exacerbations were correlated with COMCOLD scores in the present study.

Data on further exacerbations during the one-year- follow-up period were obtained, regardless of the severity of exacerbation. Comorbidities defined by the CCI and COMCOLD were correlated with further exacerbations. This result indicates that the CCI and COMCOLD indices are valuable for determining the risk of COPD exacerbation. The frequency and severity of exacerbations in COPD are related to the severity of the disease, which gradually increases with time. Based on these results, it can be concluded that comorbidity indices could play an important role in determining mortality rates. However, the COTE index was not found to be an indicator of further exacerbation. The small number of patients in the high COTE group could be the reason for this finding.

In this study, CCI, COTE, and COMCOLD scores were correlated with the frequency and severity of AECOPD. To our knowledge, this is the first study to compare the reliability of these three indices in estimating the anticipated frequency of AECOPD episodes. Further studies with larger sample sizes are required to confirm these findings.

Our study has some strengths and limitations. One of the strengths of this study is that the patients were prospectively enrolled in the study, not retrospectively, using records with ICD codes. The other strength is that every patient’s COPD diagnosis was confirmed using spirometry. The most important limitation of this study is the small number of participants. Therefore, studies with larger cohorts are required to validate these findings.

## CONCLUSION

Comorbidities are among the most important risk factors for AECOPD. The management of comorbidities begins by recognizing them. Screening for comorbidities that can affect the disease course is vital. Therefore, incorporating at least one comorbidity index during assessment ensures that comorbidities are not overlooked during diagnostic and therapeutic processes. The CCI, COTE, and COMCOLD are useful tools to obtain information about the comorbidities affecting exacerbations in COPD patients.

## ACKNOWLEDGMENTS

This study is a MECOR Turkey project of Elif Şen MD, which is a collaboration of the Turkish Thoracic Society and American Thoracic Society.

**Ethical Committee Approval:** This study was approved by the Ankara University Faculty of Medicine Clinical Research (Decision no: 05-305-18, Date: 12.03.2018).

## CONFLICT of INTEREST

The authors declare that they have no conflict of interest.

## AUTHORSHIP CONTRIBUTIONS

Concept/Design: FA, EŞ Analysis/Interpretation: DDM Data acqusition: DDM, BB, SA Writing: DDM

Clinical Revision: FA, EŞ Final Approval: All of authors

## REFERENCES

1. *
  Burge S, Wedzicha J. COPD exacerbations: Definitions and classifications. Eur Respir J 2003; 21: 46-53. https:// doi.org/10.1183/09031936.03.00078002*
2. *
  Vogelmeier CF, Criner GJ, Martínez FJ, Anzueto A, Barnes PJ, Bourbeau J, et al. Global strategy for the diagnosis, management, and prevention of chronic obstructive lung disease 2017 report: GOLD Eexecutive Summary. Arch Bronconeumol 2017; 53: 128-49. https://doi. org/10.1016/j.arbres.2017.02.001*
3. *
  Miravitlles M, Guerrero T, Mayordomo C, Sánchez-Agudo L, Nicolau F, Segú JL. Factors associated with increased risk of exacerbation and hospital admission in a cohort of ambulatory COPD patients: A multiple logistic regression analysis. The EOLO Study Group Respiration 2000; 67: 495-501. https://doi.org/10.1159/000067462*
4. *
  Donaldson GC, Wilkinson TM, Hurst JR, Perera WR, Wedzicha JA. Exacerbations and time spent outdoors in chronic obstructive pulmonary disease. Am J Respir Crit Care Med 2005; 171: 446-52. https://doi.org/10.1164/ rccm.200408-1054OC*
5. *
  Donaldson GC, Hurst JR, Smith CJ, Hubbard RB, Wedzicha JA. Increased risk of myocardial infarction and stroke fol- lowing exacerbation of COPD. Chest 2010; 137: 1091-7. https://doi.org/10.1378/chest.09-2029*
6. *
  Kiyokawa H, Muro S, Oguma T, Sato S, Tanabe N, Takahashi T, et al. Impact of COPD exacerbations on oste- oporosis assessed by chest CT scan. COPD 2012; 9: 235- 42. https://doi.org/10.3109/15412555.2011.650243
  *
7. *
  Vermeeren M, Schols A, Wouters E. Effects of an acute exacerbation on nutritional and metabolic profile of patients with COPD. Eur Respir J 1997; 10: 2264-9. https://doi.org/10.1183/09031936.97.10102264*
8. *
  Vestbo J, Anderson W, Coxson HO, Crim C, Dawber F, Edwards L, et al. Evaluation of COPD longitudinally to identify predictive surrogate end-points (ECLIPSE). Eur Respir J 2008; 31: 869-73. https://doi. org/10.1183/09031936.00111707
  *
9. *
  Burgel PR, Roche N, Paillasseur JL, Tillie-Leblond I, Chanez P, Escamilla R, et al. Clinical COPD phenotypes identified by cluster analysis: Validation with mortality. Eur Respir J 2012; 40: 495-6. https://doi. org/10.1183/09031936.00228511*
10. *
  Dahlén I, Janson C. Anxiety and depression are related to the outcome of emergency treatment in patients with obstructive pulmonary disease. Chest 2002; 122: 1633-7. https://doi.org/10.1378/chest.122.5.1633*
11. *
  Gudmundsson G, Gislason T, Janson C, Lindberg E, Hallin R, Ulrik CS, et al. Risk factors for rehospitalisation in COPD: Role of health status, anxiety and depression. Eur Respir J 2005; 26: 414-9. https://doi.org/10.1183/09031 936.05.00078504*
12. *
  Küpeli E, Ulubay G, Ulasli SS, Sahin T, Erayman Z, Gürsoy
  *

*
A. Metabolic syndrome is associated with increased risk of acute exacerbation of COPD: A preliminary study. Endocrine 2010; 38: 76-82. https://doi.org/10.1007/ s12020-010-9351-3*

13. *
  Lin Y, Tsai C, Chien L, Chiou H, Jeng C. Newly diagnosed gastroesophageal reflux disease increased the risk of acute exacerbation of chronic obstructive pulmonary disease during the first year following diagnosis-a nationwide pop- ulation‐based cohort study. Int J Clin Pract 2015; 69: 350- 7. https://doi.org/10.1111/ijcp.12501*
14. *
  Trinkmann F, Saur J, Borggrefe M, Akin I. Cardiovascular comorbidities in Chronic Obstructive Pulmonary Disease (COPD) - current considerations for clinical practice. J Clin Med 2019; 8: 69. https://doi.org/10.3390/jcm8010069
  *
15. *
  Koskela HO, Salonen PH, Romppanen J, Niskanen L. A history of diabetes but not hyperglycaemia during exacer- bation of obstructive lung disease has impact on long-term mortality: A prospective, observational cohort study. BMJ Open 2015; 5: e006794. https://doi.org/10.1136/bmjop- en-2014-006794*
16. *
  Rizkallah J, Man SP, Sin DD. Prevalence of pulmonary embolism in acute exacerbations of COPD: A systematic review and metaanalysis. Chest 2009; 135: 786-93. https://doi.org/10.1378/chest.08-1516*
17. *
  Mannino DM, Thorn D, Swensen A, Holguin F. Prevalence and outcomes of diabetes, hypertension and cardiovascu- lar disease in COPD. Eur Respir J 2008; 32: 962-9. https:// doi.org/10.1183/09031936.00012408
  *
18. *
  Cavaillès A, Brinchault-Rabin G, Dixmier A, Goupil F, Gut- Gobert C, Marchand-Adam S, et al. Comorbidities of COPD. Eur Respir Rev 2013; 22: 454-75. https://doi. org/10.1183/09059180.00008612*
19. *
  Jeong SH, Lee H, Carriere KC, Shin SH, Moon SM, Jeong BH, et al. Comorbidity as a contributor to frequent severe acute exacerbation in COPD patients. Int J Chron Obstruct Pulmon Dis 2016; 11: 1857. https://doi.org/10.2147/ COPD.S103063*
20. *
  Almagro P, Cabrera FJ, Diez J, Boixeda R, Alonso Ortiz MB, Murio C, et al. Comorbidities and short-term prognosis in patients hospitalized for acute exacerbation of COPD: The EPOC en Servicios de medicina interna (ESMI) study. Chest 2012; 142: 1126-33. https://doi.org/10.1378/ chest.11-2413*
21. *
  Charlson ME, Pompei P, Ales KL, MacKenzie CR. A new method of classifying prognostic comorbidity in longitudi- nal studies: Development and validation. J Chronic Dis 1987; 40: 373-83. https://doi.org/10.1016/0021- 9681(87)90171-8
  *
22. *
  Ofori-Asenso R, Zomer E, Chin KL, Si S, Markey P, Tacey M, et al. Effect of comorbidity assessed by the charlson comorbidity index on the length of stay, costs and mortal- ity among older adults hospitalised for acute stroke. Int J Environ Res Public Health 2018; 15: 2532. https://doi. org/10.3390/ijerph15112532
  *
23. *
  Divo M, Cote C, de Torres JP, Casanova C, Marin JM, Pinto- Plata V, et al. Comorbidities and risk of mortality in patients with chronic obstructive pulmonary disease. Am J Respir Crit Care Med 2012; 186: 155-61. https://doi. org/10.1164/rccm.201201-0034OC*
24. *
  Frei A, Muggensturm P, Putcha N, Siebeling L, Zoller M, Boyd CM, et al. Five comorbidities reflected the health status in patients with chronic obstructive pulmonary disease: The newly developed COMCOLD index. J Clin Epidemiol 2014; 67: 904-11. https://doi.org/10.1016/j. jclinepi.2014.03.005*
25. *
  Graham BL, Steenbruggen I, Miller MR, Barjaktarevic IZ, Cooper BG, Hall GL, et al. Standardization of spirometry 2019 update. An official American thoracic society and European respiratory society technical statement. Am J Respir Crit Care Med 2019; 200: e70-e88. https://doi.org/10.1164/rccm.201908-1590ST
  *
26. *
  Almagro P, Calbo E, Ochoa de Echagüen A, Barreiro B, Quintana S, Heredia JL, et al. Mortality after hospitaliza- tion for COPD. Chest 2002; 121: 1441-8. https://doi. org/10.1378/chest.121.5.1441*
27. *
  Spece LJ, Epler EM, Donovan LM, Griffith MF, Collins MP, Feemster LC, et al. Role of comorbidities in treatment and outcomes after chronic obstructive pulmonary disease exacerbations. Ann Am Thorac Soc 2018; 15: 1033-8. https://doi.org/10.1513/AnnalsATS.201804-255OC*
28. *
  Budweiser S, Harlacher M, Pfeifer M, Jörres RA. Co-morbidities and hyperinflation are independent risk factors of all-cause mortality in very severe COPD. COPD 2014; 11: 388-400. https://doi.org/10.3109/15412555.2 013.836174
  *
29. *
  Figueira Gonçalves JM, Martín Martínez MD, Pérez Méndez LI, García Bello MÁ, Garcia-Talavera I, Hernández SG, et al. Health status in patients with COPD according to GOLD 2017 classification: Use of the COMCOLD score in routine clinical practice. COPD 2018; 15: 326-33. https://doi.org/10.1080/15412555.2018.1531388
  *

